# Perceived Discrimination as a Critical Factor Affecting Self-Esteem, Satisfaction with Physical Appearance and Depression of Racial/Ethnic Minority Adolescents in Korea

**DOI:** 10.3390/bs13040343

**Published:** 2023-04-20

**Authors:** Hyemee Kim, Kwanghyun Han, Seojin Won

**Affiliations:** 1 Department of Social Welfare, Incheon National University, Incheon 22012, Republic of Korea; 2 Institute of Social Welfare, Seoul National University, Seoul 08826, Republic of Korea; 3 Department of Social Welfare, Daegu Cyber University, Gyeongsan 38453, Republic of Korea

**Keywords:** depression, perceived discrimination, ethnic minority adolescents in Korea, satisfaction with physical appearance, self-esteem, mental health

## Abstract

The effect of perceived discrimination on adolescents’ developmental outcome has long been a topic of research, however, little is known about how it affects their depression especially among the racial/ethnic minority adolescents in Asian countries. In Korea, a country with a relatively short history of immigrant influx, discrimination has become an important social issue affecting a rapidly growing population. This study examines the impact of perceived discrimination on racial/ethnic minority adolescents in Korea, specifically focusing on its impact on depression through self-esteem and satisfaction with physical appearance. The Multicultural Adolescents Panel Study data were used for analyses, and the SPSS Process Macro program was used to test the parallel mediating effects of self-esteem and satisfaction with physical appearance. The findings show that perceived discrimination was a strong predictor of their depression. Self-esteem and satisfaction with physical appearance also had significant mediating effect. There were no distinct gender differences among paths though the male adolescents were found to have more discriminatory experiences than the female adolescents. The findings call for the development of healthy coping strategies for these adolescents to prevent the effect of perceived discrimination, not only for their mental health, but also with their self-perception including physical appearance.

## 1. Introduction

Over the past decades, Korea has become a destination country for many immigrants across the globe. As of 2022, there are approximately 2,245,912 foreign-born residents in Korea, which includes short-term visitors, migrant workers, naturalized immigrants, refugees, and sojourners [1]. The number of foreign-born residents in Korea has been on a continuous rise and they currently make up about five percent of the entire Korean population [1]. With a relatively shorter history of immigrant influx and a rise of socially alarming incidents involving hate crimes and discrimination against their immigrant population, Korean society has undertaken nation-wide projects specifically targeting Korean nationals to increase and improve tolerance, acceptance, and celebration of cultural, racial, and ethnic diversity [2]. This has been an important task for Korean society as many racial/ethnic minority children, adolescents, and adults are reported to be constant victims of harassment and discriminatory actions on an everyday basis [3,4].

Adolescence is a critical period of cognitive, psychological, physical, and social development. There are many developmental tasks and issues at hand, ranging from developing a positive self-image to building healthy social relationships. Some of these tasks, however, pose challenges for many ethnic minority children and adolescents as they constantly encounter unpleasant experiences in which they are viewed, judged, and often mistreated because of their ethnicity, race, and/or immigrant background [5,6,7]. Racial and/or ethnic discrimination, a differential treatment based on one’s race and/or ethnicity, has been one of the critical factors threatening the healthy development of children and adolescents [7]. According to Garcia-Coll et al. [8], racism, discrimination, oppression, and prejudice are non-shared experiences of the minority children, and they are ‘central drivers’ [5] (p. 856) of these children’s development. Early and continuous exposure to racial and/or ethnic discrimination pave the pathways to their development, shaping and influencing the world they live in and interact with. Their immediate environment, including school, neighborhood, and family, is affected by exposure. A myriad of these factors all together influence their cognitive, social, physical, and psychological development [8].

One of the most pernicious outcomes for racial and/or ethnic minority adolescents is their poor mental health. Depression is a serious mental health issue for individuals of all ages, and it is also one of the most pressing mental health problems for adolescents [9]. According to recent statistics [10], one in seven adolescents from 10 to 19 years of age suffer from mental disorders, and depression is one of the leading mental health problems of this age group. Depression is also multifactorial [10,11] involving a multiple sets of risk factors. For racial/ethnic minority adolescents, perceived discrimination functions as a chronic stressor and a major risk factor, making them more vulnerable to depression [12,13,14]. Empirical studies testing the association between perceived discrimination and depression are in abundance, all point to a similar finding, which is that discrimination, indeed, significantly predicts adolescents’ mental health problems [3,4,5,7,12,13,14,15,16,17,18]. Benner et al. [5]’s recent work, a meta-analysis of 214 publications on adolescents and racism, attests to the finding. In their study, adolescents who reported greater perception of racial and/or ethnic discrimination were more likely to exhibit more depressive symptoms as well as poorer development outcomes in other areas such as self-esteem and social engagement.

While attempts to investigate the relationship between perceived discrimination and depression are in abundance, with a large body of empirical evidence, there are still areas that remain unknown. More specifically, the effect of perceived discrimination on their mental health needs to be explored in context of their developmental characteristics. Adolescence is a developmental period during in which their self-concept is shaped, and their perception of the self heavily relies on how they perceive, experience, and interact with their social world [5,8,19,20]. As such, it is important to take adolescent’s perception of themselves into further probing of the discrimination-depression relationship. For example, self-esteem, an important part of the self-concept, which is defined as an overall assessment of the self [20], is an important construct that must be considered in studies that examine adolescents’ mental health and psychological development. Self-esteem is an important part of the self-concept that often functions as a protective resource for adolescents, buffering the effect of various stressors on their lives [19,20]. Having a healthy regard for oneself is important for adolescents’ growth and is often identified as a strong predictor of adolescents’ depression [3,14,19,20]. However, self-esteem can be volatile and can be significantly affected by events, stressors, and/or circumstances [21,22]. For ethnic minority adolescents, one of the major risk factors in developing a healthy self-concept is their perceived discrimination [3,20,23]. Continuous negative perceptions and feedback they receive from others leads the ethnic minority adolescents to internalize such perception, stereotypes, and biases, resulting in the formation of poor self-concept [20,23]. A damaged self-concept including self-esteem increases the likelihood of experiencing depression. Kim and Won’s recent study [3] on racial/ethnic minority children and adolescents in Korea, provides supporting empirical evidence as they reported significant relationships among perceived racial/ethnic discrimination, self-esteem, and mental health outcome.

In addition to self-esteem, another potential factor that needs to be considered is racial/ethnic minority adolescents’ perception of their physical appearance. Accounting for their perception and satisfaction with their physical appearance is critical in studying racial/ethnic minority adolescents, as the stigma and discrimination they face daily is often based on their physical characteristics [24,25]. Concerns over physical appearance are also a common characteristic of adolescents, and dissatisfaction with their physical appearance reportedly affects many adolescents worldwide with potential long-term consequences that persist throughout their adulthood [26,27]. Adolescents’ satisfaction with their physical appearance is known to be linked with eating disorders, depression, anxiety, and social withdrawal [24,25,26,27,28,29,30,31,32]. There are various reasons behind such disliking of their appearances. For example, adolescents’ constant exposure to negative perception and feedback from their social world may play a role. Just as self-esteem is heavily influenced by experiences in their social world, their perception of their physical appearance can also be affected by negative experiences they encounter in their daily lives [25,31]. This is particularly the case for ethnic minority adolescents in Korea, a country known for its relatively homogeneous ethnic population. Racial/ethnic discrimination based on physical appearance is reportedly a common experience shared by many racial/ethnic minority groups [2,24,25]. Many minority school-aged children and adolescents are often the victims of aggression, violence, and emotional and verbal harassment because they ‘look different’ [4,25,32]. One study reported that 15% of racial/ethnic minority adolescents in Korea experienced some type of harassment by their peers because of their physical appearances [32].

While scholars have long argued the importance of considering adolescents’ social and contextual experiences [5,8], studies linking perceived discrimination and adolescents’ satisfaction of physical appearance are overall lacking. A large portion of studies on physical appearances including body satisfaction are focused on African American and/or mostly Latino adolescents [31,33,34]. Little is known about racial/ethnic minority adolescents’ assessment and satisfaction of their physical appearances in Asian countries, such as Korea. Furthermore, less is known about the perceived discrimination-depression relationship accounting for adolescents’ important characteristics such as self-esteem and satisfaction with their physical appearance. This study is thus the first known attempt to probe into the relationship, specifically examining the mediating roles of self-esteem and satisfaction with physical appearance. In addition, this study also investigates gender differences. Gender differences in depression, self-esteem, and satisfaction with physical appearances are well-noted in previous studies reporting a higher risk for girls than boys [14,15,17,18,28,30,35,36]. As for perceived racial/ethnic discrimination, the findings remain rather inconclusive. Few studies that examined gender differences in perceived discrimination and its impact on various outcome indices have commonly demonstrated that boys and men are reportedly more susceptible to its effect on depression [15,17,18]. Assari et al.’s study [18] on Arab Americans showed that male Arab Americans were more cognizant about their encounter with discriminatory behavior against them resulting in elevated psychological distress, while the association was not significant among female Arab Americans. The male’s susceptibility to perceived discrimination is also noted in other studies linking their experiences to substance abuse [36]. However, less is known about gender differences on the impact of discrimination on adolescents. Therefore, accounting for gender differences in this perceived racial/ethnic discrimination and adolescents’ developmental outcomes needs to be further investigated. Furthermore, such investigations in the context of an Asian country, namely Korea, would further expand the scope of literature on racial/ethnic minority adolescents and their development.

## 2. Data and Method

### 2.1. Data

We used the data from the 6th wave of Multicultural Adolescent Panel Survey [MAPS] collected and organized by the National Youth Policy Institute of Korea [NYPI]. This annual Survey began in 2011 with an aim to explore and track the development of multicultural adolescents in Korea, a racial/ethnic minority group of adolescents who are either born, raised, and/or adopted by couples of international marriages, families of immigrant background such as refugees, and migrant workers. The MAPS is a nationally representative data which draws its sample from 2537 public schools in 16 major cities of all provinces, and it collects various information from adolescents who were initially in their fourth grade of grammar school in 2011 and their foreign-born parent(s) [37]. The MAPS is the only existing data that is open for public use, it contains information on their psychosocial development and academic performance, as well as family and environmental factors including cultural and acculturation-related factors. This study uses raw data from the six waves of the data that was collected in 2016. A total of 1177 adolescents of 15 years old participated in 2016.

### 2.2. Measures

Depression: The dependent variable of this study is depression. Depression is measured using the Korean version of the ‘Symptom Checklist-90 Revision’ [38,39]. A total of 10 items are included in the scale, and responses were rated using a four-point Likert scale ranging from ‘not at all’ to ‘very much ‘. For analyses, we converted scores to have a range of 0~3, and a total score was used. A higher score indicates a more severe degree of depression. The reliability of the scale for the data, Cronbach’s alpha, was 0.904.

Perceived discrimination: The key independent variable of this study is perceived discrimination of racial/ethnic minority adolescents in Korea. The perceived discrimination scale, originally developed by Hovey and King [40], was translated and modified by Noh [41] and Hong [42] to adopt to study immigrant adolescents’ experiences in Korea. Perceived discrimination was measured using four items such as “People look down on me because my parent(s) are from another country” and “Koreans harass me and my family”. Responses were recorded on a four-point Likert scale ranging from ‘not at all (0)’ to ‘very much (3)’. The scores were summated to have a range between zero to 12. A higher score is indicative of a higher level of perceived discrimination. Cronbach’s alpha was 0.933.

Self-esteem: As one of the mediators, self-esteem measures adolescents’ overall assessment of themselves. The self-esteem scale used in the MAPS is a translated, modified version of the self-esteem scale [43] originally developed by Coopersmith [44]. The scale consists of four items asking questions such as “I am proud of myself”, “I think I am a good person”. Adolescents were asked to rate their responses on a four-point Likert scale ranging from ‘strongly disagree (0)’ to ‘strongly agree (3)’. A summated score was used for the analyses, and a higher score indicates a higher level of self-esteem for adolescents. The Cronbach’s alpha was 0.832.

Satisfaction with physical appearance: Another mediator used in this study was adolescents’ satisfaction with their physical appearance. It was measured using a six-item scale developed by Song [45] which was then modified for children and adolescents’ use by Han [46]. Adolescents were asked to rate how strongly they disagree or agree with each statement of the questionnaire, such as “I think I am physically attractive” and “I feel terrible about how I look”. Some responses were reverse coded, and a summated score was used for analyses. A higher score indicates a higher level of satisfaction with their physical appearance, the Cronbach’s alpha was 0.775.

Socio-demographic variables: Gender, household income, and adolescent’s area of residence were included. Gender was coded with one being assigned to female and zero being male. Information on monthly household income was provided by their parents. The MAPS also asks adolescents about their place of residence, and we created a binary variable indicating one for urban areas and zero for rural areas.

### 2.3. Analyses

To examine the mediating effect of self-esteem and satisfaction with physical appearance on the perceived discrimination-depression relationship, we used SPSS 23.0 and SPSS Process Macro 4.2. First, we conducted descriptive statistics with means and standard deviation of the key variables along with analyzing gender differences using t-test. Then, we calculated Pearson’s correlation coefficients among the key variables. Finally, to test for the parallel mediating effect, the Hayes’s [47] suggested method of SPSS Process Macro with bootstrapping method was employed, as it provides the direct, indirect, and total effects when there are more than one mediating variables [48].

## 3. Results

Table 1 shows the descriptive statistics of the key variables. Among 1177 adolescents in the multicultural family, 591 adolescents (50.2%) were male and 586 adolescents were (49.8%) female. The depression mean for total sample was 7.01 (SD = 5.31), while female adolescents (Mean = 7.58, SD = 5.41) tended to feel more depressed than male adolescents (Mean = 6.45, SD = 5.14). The mean of perceived discrimination score was 0.25 (SD = 0.55). However, male adolescents (Mean = 0.30, SD = 0.59) scored higher than that of female adolescents (Mean = 0.21, SD = 0.50). The total mean of self-esteem was 8.58 (SD = 2.18). Male adolescents (Mean = 8.65, SD = 2.12) scored slightly higher on their self-esteem scale than female adolescents (Mean = 8.51, SD = 2.23), though the difference was not statistically significant. The mean of satisfaction of physical appearance for total sample was 12.18 (SD = 2.95). Male adolescents (Mean = 12.41, SD = 2.81) were more satisfied with their physical appearance than female adolescents (Mean = 11.96, SD = 3.08).

Correlations among variables included in the research model are indicated in Table 2 below.

The results of testing mediation effects are shown in Table 3. The results revealed that perceived discrimination negatively impacted on self-esteem (B = −0.89, *p* < 0.001) and satisfaction of physical appearance (B = −1.17, *p* < 0.001). In addition, perceived discrimination increased the level of depression (B = 1.31, *p* < 0.001), showing that the more they are exposed to discrimination, the higher their depression score is. Self-esteem (B = −0.66, *p* < 0.001) and satisfaction of physical appearance (B = −0.44, *p* < 0.001) had negative association with depression. In other words, adolescents with higher self-esteem and stronger satisfaction with their physical appearances are less likely to be depressed than those with lower self-esteem and those who are less satisfied with how they look.

The mediation analyses showed that the size of total effect of perceived discrimination on depression reduced to direct effect when self-esteem and satisfaction of physical appearance (B = 2.40, *p* < 0.001 → B = 1.31, *p* < 0.001) were accounted for. Perceived discrimination had a statistically significant effect on self-esteem and self-esteem also significantly affected depression. Likewise, perceived discrimination had a significant relationship with satisfaction of physical appearance, and satisfaction of physical appearance also significantly predicted depression. With all the conditions met for mediating testing analysis, the indirect effect was assessed. The result is shown in Table 4.

**Figure 1:**
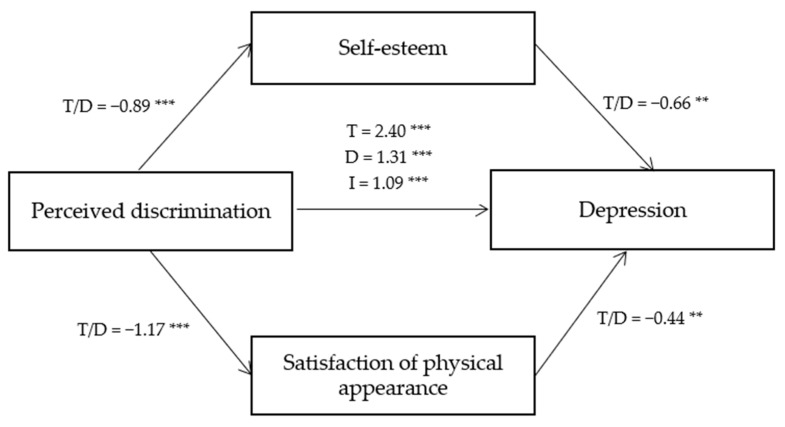
Mediation effects of self-esteem and satisfaction of physical appearance. * *p* < 0.05, ** *p* < 0.01, *** *p* < 0.001. T = Total effect, D = Direct effect, I = Indirect effect.

The paths analyses results among perceived discrimination, depression, self-esteem, and satisfaction of physical appearance are shown in Figure 1 below. 

As shown in Table 4, indirect effects of self-esteem and satisfaction of physical appearance were statistically significant as both LLCI and ULCI did not include 0.

Gender differences were then examined. Separate path analyses were conducted for male and female adolescents. The results are shown in Table 5. Both male and female models had similar results to that of the whole sample model. Perceived discrimination in both groups had significant effect on their depression (males, B = 1.35, *p* < 0.001; females, B = 1.25, *p* < 0.001) as it affected both their self-esteem and satisfaction with physical appearance. For example, for male adolescents, perceived discrimination was negatively associated with their self-esteem (B = −0.97, *p* < 0.001) and satisfaction of physical appearance (B = −1.35, *p* < 0.001). As in the analysis with the whole sample, self-esteem (B = −0.76, *p* < 0.001) and satisfaction of physical appearance (B = −0.43, *p* < 0.001) were both negatively associated with their depression. Male adolescents who are less satisfied with their physical appearance and had lower self-esteem reported a higher level of depression. The mediating effects of both self-esteem and satisfaction with physical appearance were also present in the male adolescent sample as well.

Similarly, perceived discrimination of the female minority adolescents decreased their self-esteem (B = −0.78, *p* < 0.001) and their satisfaction of physical appearance (B = −0.92, *p* < 0.001). Self-esteem (B = −0.56, *p* < 0.001) and satisfaction of physical appearance (B = −0.45, *p* < 0.001) were negatively associated with female adolescents’ depression, same as in the results of the male adolescents’ sample. Same as the male model, in the female model, the total effect decreased after adding the two mediating variables in the model (B = 2.10, *p* < 0.001 → B = 1.25, *p* < 0.001), indicating potential mediating effect of the two variables. To test the significance of the mediation effect of two variables, bootstrapping analyses were conducted. As shown in Table 6, the indirect effect of both self-esteem and satisfaction with physical appearance were significant.

Significant gender variation was not observed. The paths among perceived discrimination leading to depression were found equally significant for both male and female minority adolescents. For both groups, perceived discrimination was the strongest predictor of depression followed by self-esteem and physical appearances. However, for female adolescents, their place of residence also had significant association with their depression. Those who live in urban areas were more likely to be depressed than the girls in rural areas, whereas it did not have significant predicting power of male adolescents’ depression.

## 4. Discussion

This study investigated the perceived discrimination-depression relationship accounting for adolescents’ characteristics namely self-esteem and satisfaction with physical appearance in Korea, a country with a relatively shorter immigrant history. Adding to the body of literature on discrimination-depression relationships [3,4,5,6,7,12,13,14,15,16,17,18,20,23], the findings of this study suggest that adolescents’ perceived discrimination is, indeed, the strongest predictor of their depression. Despite the low degree of exposure to discrimination reported by the adolescents, its impact on their mental health remains quite strong accounting for the effect of other variables. This finding indicates that the racial/ethnic minority adolescents in Korea are at a high risk of developing mental health difficulties including depression as they are continuously exposed to everyday experiences of discrimination perpetuated by their peers, teachers, neighbors, and people in general. Adolescents are more vulnerable to harmful effects of discrimination because adolescence is the developmental period when they become more cognizant of themselves in context of their environment [5,7]. Early exposure to negative perceptions and behaviors from others may also leave the adolescents with a poor self-concept, social, and coping skills because their cognitive and social developments are still in process. This finding calls for a strong need to assist these adolescents and their families to develop healthy coping skills in addressing their discrimination experiences.

The results also suggest that self-esteem and adolescents’ satisfaction with their physical appearance both play critical roles in mediating the effect of perceived discrimination on depression. The finding that perceived discrimination indeed hurts adolescents’ self-esteem, is in line with that of previous studies [3,14,20,21,23]. The finding also supports what we know about the relationship between self-esteem and depression [3,9,14,19,23]. The significant association among the variables is alarming given that self-esteem is an important self-construct which can also function as a buffer for stressors [19,20], and lower self-esteem is also linked to negative outcomes such as poor academic performance, social relationships, and lower life satisfaction [19,20,21,22,23,24]. This finding suggests that scholars and practitioners who work with minority adolescents address their self-esteem in relation to their discrimination experiences in-depth. For instance, there are numerous programs offered to racial/ethnic minority children and adolescents at a community level in Korea. However, a large majority of them evolve around language assistance, cultural education, and school-work related activities [2]. Only a handful of programs are specifically designed to improve their self-esteem [2,49,50]. Provided in forms of group programs or group therapy, they mainly work with racial/ethnic minority children and adolescents to increase their overall self-esteem. However, these programs rarely address their everyday experiences as marginalized members of society, and most of the programs do not adequately deliver helpful content on coping and dealing with such incidents. It is therefore suggested that school and community programs, assisting racial/ethnic minority adolescents and their families, develop and implement programs to address their self-esteem needs, considering their experiences as minorities in Korean society. Furthermore, an emphasis on building strong ethnic identity also needs to be given, as having strong ethnic identity functions as a protective factor for many racial/ethnic minority adolescents [51].

A noteworthy finding is the mediating role of adolescents’ satisfaction with their physical appearances. Adolescents’ perception of their body image and physical appearance has long been a topic of interest for many scholars [24,25,26,27,28]. However, understanding of racial/ethnic minority adolescents’ perception of their own physical appearances, in association with their perceived discrimination, is still lacking. The finding that perceived discrimination of racial/ethnic minority adolescents in Korea significantly affects their satisfaction with physical appearance, is an important addition to the body of literature. As adolescents’ perception of themselves is greatly shaped by how they believe others perceive them, and their belief or view is often reinforced by their personal experiences within their social world. It is likely that negative experiences they encounter, being a racial/ethnic minority, not only affect their appraisal of their self-worth, but also how they physically look. Racial/ethnic minority adolescents often face challenges in forming and maintaining a healthy sense of self in a society that views them as different, abnormal, or “deviant” [34] (p. 619) from the normal or ideal appearance. Korean society upholds fair-skin, large eyes, pointy nose, and slim figures as ideal and beautiful, and the minority adolescents who are visibly darker and less similar to the ideal image are often the targets of verbal harassment and unfair treatment [52]. As a cause of an accumulation of negative experiences with Korean peers, neighbors, and the public in general, the findings indicate that these minority adolescents are likely to be dissatisfied with their own body and physical characteristics. How they perceive their own body and appearance becomes an important component of their identity, and it is reported that how they look can also influence their self-esteem and vice versa [25,30,53]. The findings of this study indicate that along with poor self-esteem that may also be a byproduct of perceived discrimination, the adolescents may also be left with distorted perceptions of their own body and appearance. Damaged self-image, as shown in this study, also hurts their mental health, leading to the conclusion that the effect of perceived discrimination may be more harmful than what is known to this date.

The findings of the study also shed light on the importance of measures and effort to eliminate and/or reduce discriminatory behavior by the public in general. While the development and implementation of various intervention tools may be useful to assist minority adolescents to cope with such events, it is even more critical to foster a social environment in which diversity is accepted and celebrated. Discrimination based on race and/or ethnicity is often deeply rooted in the social structure, and education is a key component in increasing public awareness, ultimately eradicating discrimination [54]. A mere incorporation of diversity education sessions may not be adequate in tackling the issue, and further steps must be taken in education and education institutions. Examples of changes in the education system include: restructuring of curriculum to reflect, incorporate, and celebrate diversity, as well as providing training and education seminars to policy makers, teachers, administrators, and students on their own implicit bias and discriminatory behavior [54]. School curriculums need to be examined extensively to eliminate any contents that may depict or insinuate racist or biased depictions and representations of racial/ethnic groups. More intensive and age-appropriate education sessions must be designed and provided to assist all students and faculties to be aware of their own bias and how their bias and behaviors may affect minority groups as shown in this study.

Significant gender differences in paths leading to depression were not observed in this study. The effect of perceived discrimination on depression was equally strong for both male and female adolescents, though the effect was slightly stronger for male adolescents. Being one of the few studies examining gender differences in perceived discrimination literature, the finding that both male and female adolescents are heavily impacted by perceived discrimination via lowered self-esteem and satisfaction with physical appearance provides an important insight for their development. The finding shows that perceived discrimination is indeed a strong threat for all adolescents’ development, and it can affect their self-perception. Though gender differences were not visible in paths among the variables, descriptive statistics show that male adolescents indeed had higher scores on perceived discrimination than the female adolescents, consistent with reports of previous studies [15,17,18,36]. Male adolescents’ higher cognizance of discrimination can be explained by the subordinate male target hypothesis [53]. According to the hypothesis, male racial/ethnic minorities are considered more of a threat than the females by the mainstream society, particularly by the males of the dominant group. Though the hypothesis remains quite controversial to this date, the finding of this study warrants attention for more in-depth understanding of the gendered nature of discrimination. As discrimination is multi-faceted, future studies need to examine how different forms of discrimination take place and whether gender variation is observed in different age groups of the minority population.

In sum, this study provides important empirical evidence to discrimination-mental health research. The findings of the study revealed that the ethnic minority adolescents’ sense of themselves is deeply damaged by perceived discrimination which may play a role in deterioration of mental health. Furthermore, this study focuses on racial/ethnic minority adolescents in Korea, a country with a relatively shorter immigrant history. While the number of racial/ethnic minority population is growing steadily, society’s perception towards immigrants and people with immigrant background remains hostile. As adolescents’ development heavily relies upon their experiences, not only with their immediate social world, but also with society at large, it is important to understand the effect of their perceived discrimination on their development. The findings of the study call for a societal level intervention including the restructuring of the education system, curriculum, and the development and provision of programs, intervention seminars, and counseling sessions for the effected adolescents as well as the public.

## 5. Limitation

While this study provides additional and important empirical evidence to perceived discrimination-mental health literature for racial/ethnic minority populations, the study also carries several limitations. First, the cross-sectional nature of the analysis limits our understanding of the causal relationship among the variables. Future studies should consider the long-term impact of perceived discrimination on adolescents’ self-esteem and their appearance satisfaction, as well as their depression. Second, more comprehensive measures need to be used to capture adolescents’ experiences of discrimination. We only used a few items to measure their general experiences. More extensive and situation-specific measurements of perceived discrimination would further our understanding of its impact. The MAPS we used for this study also measures depression using the Symptoms Checklist [38,39]. Utilizing a more clinical scale such as CES-D would yield more information on adolescents’ depression status. Third, the relationship between self-esteem and satisfaction with physical appearance needs to be further investigated. Adolescents’ perception of their own body and physical characteristics may be in part influenced by their self-esteem and vice versa [52]. Future studies need to explore the reciprocal nature of the relationship.

The potential reciprocal relationship accounting for gender differences also needs to be examined using longitudinal data. Future studies should also investigate various coping strategies and mechanisms used by racial/ethnic minority adolescents in dealing with perceived discrimination. While there are several studies investigating the impact of discrimination on adolescents’ substance abuse [36] and eating disorders [35], there are no existing literature investigating the topic in Korea, and little is known about other coping methods and strategies employed by the adolescents and their families. Identifying effective coping strategies would provide an important clinical implication for practitioners working with minority adolescents.

## Figures and Tables

**Table 1:** Descriptive statistics of the key variables.

		Mean	SD	*t*-Test
Depression	Total	7.01	5.31	
	Male	6.45	5.14	3.68 ***
	Female	7.58	5.41	
Perceived discrimination	Total	0.25	0.55	
	Male	0.30	0.59	−2.86 **
	Female	0.21	0.50	
Self-esteem	Total	8.58	2.18	
	Male	8.65	2.12	−1.15
	Female	8.51	2.23	
Satisfaction of physical appearance	Total	12.18	2.95	
	Male	12.41	2.81	−2.62 **
	Female	11.96	3.08	

* *p* < 0.05, ** *p* < 0.01, *** *p* < 0.001.

**Table 2:** Correlations matrix of the study variables.

	1	2	3	4	5	6	7
1. Depression	1.00						
2. Perceived discrimination	0.24 ***	1.00					
3. Self-esteem	−0.45 ***	−0.22 ***	1.00				
4. Satisfaction of physical appearance	−0.45 ***	−0.21 ***	0.63 ***	1.00			
5. Gender	0.11 ***	−0.08 **	−0.03	−0.08 **	1.00		
6. Residence	0.09 **	0.03	−0.03	−0.05	−0.01	1.00	
7. Monthly household income	0.01	−0.04	0.14 ***	0.07 *	−0.04	0.13 ***	1.00

* *p* < 0.05, ** *p* < 0.01, *** *p* < 0.001.

**Table 3:** Process Macro results.

	B	SE	t	LLCI ^a^	ULCI ^b^
Model 1 (Dependent variable: Self-esteem)					
Constants	8.42 ***	0.19	45.22	8.06	8.79
Perceived discrimination	−0.89 ***	0.11	−7.80	−1.11	−0.66
Gender	−0.19	0.12	−1.54	−0.43	0.05
Residence	−0.21	0.14	−1.50	−0.47	0.06
Monthly household income	0.00 ***	0.00	4.61	0.00	0.00
Model 2 (Dependent variable: Satisfaction of physical appearance)					
Constants	12.56 ***	0.25	49.36	12.06	13.06
Perceived discrimination	−1.17 ***	0.16	−7.53	−1.47	−0.87
Gender	−0.52 **	0.17	−3.06	−0.85	−0.19
Residence	−0.33	0.19	−1.76	−0.70	0.04
Monthly household income	0.00 *	0.00	2.20	0.00	0.00
Model 3 (Dependent variable: Depression)					
Constants	15.98 ***	0.74	21.50	14.52	17.44
Perceived discrimination	1.31 ***	0.25	5.17	0.81	1.80
Self-esteem	−0.66 ***	0.08	−8.29	−0.81	−0.50
Satisfaction of physical appearance	−0.44 ***	0.06	−7.56	−0.55	−0.32
Gender	0.95 ***	0.27	3.55	0.43	1.48
Residence	0.74 *	0.30	2.49	0.16	1.32
Monthly household income	0.00 *	0.00	2.32	0.00	0.01

^a^ Low Limit Confidence Interval = Lower bound within the 95% confidence interval of the boot indirect effect. ^b^ Upper Limit Confidence Interval = Upper bound within the 95% confidence interval of the boot indirect effect. * *p* < 0.05, ** *p* < 0.01, *** *p* < 0.001.

**Table 4:** Bootstrapping results.

	B	SE	BootLLCI ^a^	BootULCI ^b^
Total indirect effect	1.09	0.13	0.85	1.35
Self-esteem	0.58	0.10	0.40	0.78
Satisfaction of physical appearance	0.51	0.10	0.34	0.72

^a^ Low Limit Confidence Interval = Lower bound within the 95% confidence interval of the boot indirect effect. ^b^ Upper Limit Confidence Interval = Upper bound within the 95% confidence interval of the boot indirect effect.

**Table 5:** Process macro results by gender.

	Male					Female				
	B	SE	t	LLCI ^a^	ULCI ^b^	B	SE	t	LLCI ^a^	ULCI ^b^
Model 1 (Dependent variable: Self-esteem)										
Constants	8.36 ***	0.23	36.01	7.91	8.82	8.28 ***	0.26	32.35	7.78	8.79
Perceived discrimination	−0.97 ***	0.14	−6.77	−1.25	−0.69	−0.78 ***	0.18	−4.25	−1.14	−0.42
Residence	−0.10	0.19	−0.55	−0.47	0.27	−0.29	0.20	−1.46	−0.69	0.10
Monthly household income	0.00 ***	0.00	3.52	0.00	0.00	0.00 **	0.00	2.97	0.00	0.00
Model 2 (Dependent variable: Satisfaction of physical appearance)										
Constants	12.57 ***	0.31	40.86	11.97	13.18	12.01 ***	0.36	33.49	11.31	12.72
Perceived discrimination	−1.35 ***	0.19	−7.09	−1.72	−0.97	−0.92 ***	0.26	−3.57	−1.42	−0.41
Residence	−0.35	0.25	−1.40	−0.84	0.14	−0.28	0.28	−1.00	−0.83	0.27
Monthly household income	0.00	0.00	1.93	0.00	0.00	0.00	0.00	1.21	0.00	0.00
Model 3 (Dependent variable: Depression)										
Constants	17.22 ***	1.02	16.94	15.22	19.21	15.73 ***	1.02	15.42	13.72	17.73
Perceived discrimination	1.35 ***	0.32	4.23	0.73	1.98	1.25 ***	0.41	3.08	0.45	2.05
Self-esteem	−0.76 ***	0.11	−6.83	−0.97	−0.54	−0.56 ***	0.11	−4.96	−0.78	−0.34
Satisfaction of physical appearance	−0.43 ***	0.08	−5.12	−0.59	−0.26	−0.45 ***	0.08	−5.57	−0.61	−0.29
Residence	0.18	0.40	0.45	−0.61	0.97	1.28 **	0.44	2.94	0.43	2.14
Monthly household income	0.00	0.00	1.48	0.00	0.01	0.00	0.00	1.89	0.00	0.01

^a^ Low Limit Confidence Interval = Lower bound within the 95% confidence interval of the boot indirect effect. ^b^ Upper Limit Confidence Interval = Upper bound within the 95% confidence interval of the boot indirect effect. * *p* < 0.05, ** *p* < 0.01, *** *p* < 0.001.

**Table 6:** Bootstrapping results by gender.

	Male				Female			
	B	SE	LLCI ^a^	ULCI ^b^	B	SE	LLCI ^a^	ULCI ^b^
Total indirect effect	1.31	0.17	1.01	1.66	0.85	0.20	0.48	1.26
Self-esteem	0.73	0.13	0.49	1.01	0.44	0.13	0.20	0.73
Satisfaction of physical appearance	0.58	0.14	0.32	0.86	0.41	0.14	0.17	0.71

^a^ Low Limit Confidence Interval = Lower bound within the 95% confidence interval of the boot indirect effect. ^b^ Upper Limit Confidence Interval = Upper bound within the 95% confidence interval of the boot indirect effect.

## Data Availability

Not applicable.
